# Perceived Success After Participation in the Summer Health Professions Education Program

**DOI:** 10.1001/jamanetworkopen.2023.52440

**Published:** 2024-01-26

**Authors:** Patricia Xirau-Probert, Tram Lai, Erik Black, Dany Fanfan, Amy Blue, Caronne C. Rush, Rachel Powers, Jeanne-Marie R. Stacciarini

**Affiliations:** 1 College of Dentistry, University of Florida Gainesville; 2 College of Nursing, University of Florida Gainesville; 3 College of Public Health and Health Professions, University of Florida Gainesville; 4 Office of Interprofessional Education, University of Florida, Gainesville

## Abstract

**Importance:**

The number of active health care professionals has not kept pace with the increasing number of minoritized individuals in the US. The Summer Health Professions Education Program (SHPEP) was developed to alleviate this underrepresentation in the health workforce.

**Objective:**

To evaluate students’ changes in perceived barriers and motivators for entering and succeeding in professional school after SHPEP participation.

**Design, Setting, and Participants:**

For this cohort study, anonymous electronic surveys were sent to the 2017 to 2021 SHPEP participants at an academic health center at a large university in the southern US. Participants were first- and second-year undergraduates interested in the health professions and enrolled in the SHPEP. Program participants were invited to complete the study.

**Exposures:**

An anonymous electronic survey was administered before and immediately after program completion.

**Main Outcomes and Measures:**

The SHPEP Career Barriers Survey (SCBS) is composed of 22 questions on motivators and 20 questions on barriers to entering and succeeding in health professional school. Students responded using a 5-point Likert scale, with 1 indicating strongly disagree and 5 indicating strongly agree. Mixed analysis of variance was used to analyze the program’s latent factors.

**Results:**

Of all 402 SHPEP participants (mean [SD] age, 19.32 [0.88] years) from 2017 to 2021, 325 completed the preprogram survey and 259 also completed the postprogram survey. Of the 325 initial participants, 4 identified as American Indian or Alaska Native, Native Hawaiian, or Pacific Islander (1.2%); 12 as Asian (3.7%); 188 as Black (57.8%); 95 as Hispanic or Latino (29.2%); 7 as White (2.2%); and 16 as multiracial (4.9%). Two hundred twelve participants were female (65.2%), and 226 were first-generation college students (69.5%). Results of the SCBS indicate that the SHPEP had a significant small to moderate association on perceived motivators (mean [SD] *x̅* = 84.60 [9.67] vs 80.95 [8.93]; *P* = .001) and decreases in perceived barriers (mean [SD] *x̅* = 48.02 [13.20] vs 51.72 [11.39]; *P* = .008). There was no significant difference in program success between studied years.

**Conclusions and Relevance:**

In this cohort study, the SHPEP appeared to provide essential support for underrepresented students as measured by improved perceived motivators and reduced perceived barriers to entering professional education. Knowledge from this study can assist educators and health care professionals who wish to implement similar enrichment programs.

## Key Points

**Question:**

Is participation in the Summer Health Professions Education Program associated with changes in perceptions of undergraduate students’ motivators and barriers to entering and succeeding in a health professional school?

**Findings:**

This cohort study of the 2017 to 2021 Summer Health Professions Education Program participants found that the program has a significant small to moderate association on perceived barriers and motivators. On average, participants’ perceived motivators significantly increased and their perceived barriers significantly decreased.

**Meaning:**

The positive associations of the Summer Health Professions Education Program with students’ perceived barriers and motivators may inform educators and health care professionals in implementing similar enrichment programs.

## Teaser

This cohort study examines whether participation in the Summer Health Professions Education Program was associated with changes in undergraduate students’ perceptions regarding motivators and barriers to entering and succeeding in a health professional school.

## Introduction

The US Supreme Court ruled a landmark decision on June 29, 2023, to effectively strike down affirmative action and prevent universities from accounting for race in college admissions.^1^ Many states are advancing and implementing antidiversity laws across the country.^2,3,4^ This weaponization of diversity initiatives exacerbates barriers for underrepresented students (those with backgrounds that are historically and disproportionately less represented in a field than the overall population) in the health professions, negatively influencing the future of health care and the pursuit of health equity. Although an increase in diverse population representation among health care professionals is crucial to promote patient-physician relationships with high satisfaction,^5^ the number of active health care students and professionals has not kept pace with the increasing number of minoritized individuals in the population.^6,7,8,9^

The 2003 Institute of Medicine’s landmark study, “Unequal Treatment,”^10^ documented lower health care quality and higher rates of illness, disability, and premature deaths among underrepresented populations in the US. The report identified increasing the number of underrepresented health professionals as a possible key strategy to eliminate health disparities.^10^ However, underrepresented students face unique challenges when pursuing health professional careers, leading to lower achievements and opportunities. These challenges include institutional discrimination^11,12^; differential treatment; low socioeconomic status; attending institutions with limited resources^13^; lack of meaningful relationships with health professional role models^11,14^; and self-doubt, stereotype threat, and pressure to hide their cultures.^13,14,15^

Summer enrichment and pathway programs for prehealth undergraduate and graduate students have been developed to address this underrepresentation. Pathway programs offer scholars academic and professional support infrastructures. Literature on summer enrichment programs for undergraduate students indicates several positive results. A meta-analysis of 16 summer programs for first-year science, technology, engineering, and mathematics students revealed a moderate positive influence on program participants’ grade point average and retention.^16^ Other positive impacts of pathway programs include improvements to participants’ academic and research knowledge, skills, and self-efficacy.^17,18,19^ These programs may also solidify professional identities and attitudes.^17,20,21^ Summer programs also demonstrate high rates of matriculation and graduation from medical schools.^22^

As part of an effort to reduce health disparities, the Robert Wood Johnson Foundation (RWJF), in partnership with academic health centers (AHCs) across the US, has sponsored a cost-free 6-week summer enrichment program for prehealth undergraduates since 1989. Originating at 6 sites, the program has since expanded to 12 AHCs across the US. Since its inception in 2016, the Summer Health Professions Education Program (SHPEP) has served more than 30 000 students as of 2023.^23^ The SHPEP aims to prepare underrepresented students to become competitive applicants to health professional schools while diversifying the health care workforce and building a culture of health.^23,24^ There is limited literature on the SHPEP’s recent outcomes; however, data collected from the 2006 to 2015 cohorts found that 64.4% of program scholars applied to dental schools. Of those who applied, 69.8% enrolled and matriculated, and 60% graduated as of 2015.^25^ Additionally, of the participants who remained in the health tracks, 8% were more likely to apply to dental and medical schools, and 10% were more likely to matriculate than nonparticipants in the comparison group.^24^ Another study evaluating the SHPEP administered at 3 AHCs found that the program contributed to reduction in students’ negative stereotypes (eg, academic ability, professional competency, and interpersonal ability) for different health professionals.^26^ Students from underrepresented backgrounds, first-generation college graduates, and those who attended the Summer Medical and Dental Education Program (predecessor of the SHPEP) or other summer college enrichment programs were more likely to report sustained intention to practice in underserved areas compared with those who did not participate in enrichment programs.^27^

Few programs focus on interprofessional education for undergraduate students in prehealth tracks. Limited literature also evaluates the SHPEP’s or other enrichment programs’ influence on perceived barriers and motivators to enter health professional schools. The purpose of this study is to describe the SHPEP and to evaluate scholars’ changes in perceived barriers and motivators, one of multiple aspects that influence underrepresented students’ success. This understanding can assist pathway programs in alleviating barriers and promoting motivators for aspiring future health care professionals, encouraging underrepresented students to pursue graduate school and thus further ensuring the success of their participants in health professions programs. Thus, the main question for this study is whether participation in the SHPEP is associated with changes in perceptions of undergraduate students’ motivators and barriers to entering and succeeding in a health professional school.

## Methods

This retrospective cohort study analyzed 5 years of the SHPEP Career Barriers Survey (SCBS) data collected from participants enrolled in the SHPEP at a large southern university (University of Florida) from academic years 2017 to 2021. The survey was administered via Qualtrics in a pre-post format, immediately before and after program completion. Self-reported demographic information, including age, race, ethnicity, and sex, was collected to characterize the students toward evaluating the SHPEP program. Informed consent was included in the Qualtrics survey. The study was approved by the University of Florida Institutional Review Board and follows the Strengthening the Reporting of Observational Studies in Epidemiology (STROBE) cohort study guideline.

### SHPEP Eligibility

The SHPEP at the University of Florida adheres to the guidelines outlined by the RWJF, which give participants the tools to successfully apply to health professional schools by (1) enriching basic science foundation; (2) providing education on financial literacy, learning skills, health policy, and a range of health care professions; (3) engaging in interdisciplinary learning opportunities to promote personal and professional development; and (4) immersing in simulation laboratory and clinical shadowing experiences. The SHPEP at the University of Florida targets undergraduate students from schools across the US with a high population of underrepresented students (eg, historically Black college and universities). Applicants select 1 of 4 pathways offered at the institution: medicine, dentistry, pharmacy, or public health. They must also meet the eligibility criteria set by the RWJF: high school graduate; current freshman or sophomore at a postsecondary institution; a minimum grade point average of 2.5; and US citizen, permanent resident, or possession of Deferred Action for Childhood Arrivals federal status. The total number of program participants from 2017 to 2021 was 402.

### SHPEP’s Academic Enrichment

The goal of the SHPEP academic enrichment program, as defined by the university’s AHCs following RWJF principles, is to promote lifelong learning by (1) equipping students with the basic science principles that underlie biomedical theory and practice; (2) offering foundational knowledge, skills, and opportunities to analyze clinically relevant problems; and (3) developing research skills and familiarity with scientific literature. Scholars dedicate 12 hours weekly to academic programming, which includes interactive lectures, readings, assignments, interprofessional research activities, and hands-on labs (eg, gene diagnostics, dissection, and simulation).

### Workshops and Seminars

Workshops and seminars throughout the SHPEP cover a wide range of topics to encourage personal and professional growth and reduce common barriers to entering and pursuing health professional education. These topics include financial literacy, interview and resume skills, personal statement critiques and admission application preparation, exposure to health career paths (eg, arts in medicine, pharmacogenics, and translational research), social determinants of health, and wellness tools.

### Career Pathway Exploration

In addition to all-cohort academic lectures and workshops, scholars dedicated 3 hours per week to their chosen career pathway, immersing them in their selected health care fields and networking with students, staff, and faculty. These activities include cardiopulmonary resuscitation simulation on dummies with the College of Medicine, teeth waxing and clinical shadowing with the College of Dentistry, public water treatment with the College of Public Health and Health Professions, and compounding lab with the College of Pharmacy. Scholars are also exposed to health professions outside the 4 main pathways via workshops and simulation laboratory experience to foster a holistic understanding of the health disciplines and explore fields they may not have considered (eg, nursing, physician assistant studies, and veterinary medicine).

### Interprofessional Education Experience

Interprofessional education experience aims to foster interdisciplinary collaboration among scholars, mirroring that of the wider health care field. Throughout the program, scholars work together on projects, such as anatomy lab and cancer treatment poster presentations, culminating in a case project competition. In this capstone activity, based on the national CLARION competition model,^28^ teams of scholars present a solution to a real-life public health issue to peers and program faculty. These presentations spark thought-provoking discussions and showcase the value of an interprofessional approach to health care.

### Mentoring

Each scholar is matched with a graduate student from their field of interest who serves as a peer mentor. Mentors coordinate shadowing and research experience, foster faculty connections, and provide interpersonal support and other salient resources.

### Personal Growth and Mental Health

Personal growth and mental health awareness are actively promoted through wellness initiatives, such as the teaching of self-care strategies and mindfulness practices (eg, breath work and guided meditation). Participants also have access to the university’s recreation facilities, as well as counseling and crisis resources. Additionally, social programming is offered outside academics to foster collaboration, relationship building, and professional networking.

### Virtual Programs

During the summer of 2020 and 2021, the SHPEP was delivered through asynchronous learning modules due to the COVID-19 pandemic. Clinical experiences were conducted virtually through guided online instruction and delivering lab kits to students’ homes (ie, pharmacy compounding lab and dental waxing lab). During this time, mental health and wellness concerns were addressed by offering daily guided meditation, crisis response training, and information on available support services.

### Program Evaluation

The SHPEP is evaluated using the SCBS, which is composed of 22 questions on “motivators to entering/succeeding in health profession school” and 20 questions on “barriers to entering/succeeding in health profession school.” The SCBS uses the 5-point Likert scale, with 1 indicating strongly disagree and 5 indicating strongly agree. Motivator and barrier questions encompassed knowledge of materials and experience required for graduate schools, academic and social support, perceived pressure, and financial concerns. Participants completed the survey via Qualtrics before and after the program. The SCBS also includes 32 questions related to demography, extracurriculars, work, and knowledge of the graduate school enrollment process. The SCBS demonstrated sufficient internal consistency,^29^ with a Cronbach α for motivator items of 0.81 for preprogram and 0.82 for postprogram; the Cronbach α for barriers was 0.87 and 0.91 for postprogram barriers. This survey is an adapted version of the 24-item Perceived Barriers to Education and Career of McWhirter et al.^30^ We adapted questions to better fit a health professions context.

### Statistical Analysis

Descriptive statistics were used to explore student-level characteristics, and inferential statistics (eg, mixed analysis of variance and correlations) were used to examine the difference within program cohorts and identify associations among student-level characteristic variables and self-reported barriers and motivators before and after participating in the SHPEP. Cohen *d* statistics were used to calculate the effect size associated with the summer program. Data were analyzed using SPSS PASW, version 25 (IBM Corp) and RStudio, version 2022 (RStudio Team).^31^ A 2-sided *P* < .05 was considered to be statistically significant.

## Results

### Participants Demographics

Of the total 402 participants during 5 years of SHPEP administration, 325 completed the preprogram survey and 259 also completed the postprogram survey, meaning data were complete for 259 students for this study (64% response rate). Of the initial 325 students, 4 (1.2%) were American Indian or Alaska Native, Native Hawaiian, or Pacific Islander; 12 (3.7%) were Asian; 188 (57.8%) were Black; 95 (29.2%) were Hispanic or Latino; 7 (2.2%) were White; and 16 (4.9%) were multiracial. Race and ethnicity was unknown or undisclosed for 3 participants (0.9%). A total of 212 participants identified as female (65.2%), and 111 as male (34.2%); sex was unknown or undisclosed for 2 (0.5%). A total of 147 (45.2%) students had been enrolled in college for less than 2 years, and 226 (69.5%) identified as first-generation college students (coming from households where care providers have less than a 4-year college degree). Two hundred six participants (63.4%) were enrolled in a public state college or university, and 35 (10.8%) were enrolled in a community or 2-year college. Although scholars were encouraged to take the SCBS, not all participants completed the survey. The number of completed surveys in 2020 and 2021 was lower than the previous years (Table 1), likely due to the online format of the program. Table 2 details SHPEP participant demographics from 2017 to 2021.

**Table 1.** SHPEP Career Barriers Survey Completion by the 2017 to 2021 SHPEP Cohorts at a Southern University

Year	Preprogram responses, No.	Postprogram responses, No.	Total responses, No. (%)
2017	79	75	80 (94.9)
2018	92	86	80 (93.5)
2019	65	51	77 (78.5)
2020	57	25	79 (43.9)
2021	32	22	86 (68.8)
Total	325	259	402

Abbreviation: SHPEP, Summer Health Professions Education Program.

**Table 2.** Participant Demographics, 2017-2021 (Based on Survey Responses)

Demographic	No. (%) of participants by year					Total No. (%) of participants
	2017	2018	2019	2020	2021	
Respondents by year	79 (24.3)	92 (28.3)	65 (20)	57 (17.5)	32 (9.8)	325 (100)
Race and ethnicity						
American Indian or Alaskan Native, Native Hawaii, or Pacific Islander	2 (50)	1 (25)	0	0	1 (25)	4 (1.2)
Asian	0	0	5 (41.7)	4 (33.3)	3 (25)	12 (3.7)
Black	54 (28.7)	61 (32.4)	32 (17)	33 (17.6)	8 (4.3)	188 (57.8)
Hispanic or Latinx	19 (20)	25 (26.3)	20 (21)	17 (17.9)	14 (14.7)	95 (29.2)
Multiracial	2 (12.5)	5 (31.2)	4 (25)	3 (18.8)	2 (12.5)	16 (4.9)
White	2 (28.6)	0	2 (28.6)	0	3 (42.9)	7 (2.2)
Prefer not to disclose or unknown	0	0	2 (66.6)	0	1 (33.3)	3 (0.9)
Sex						
Male	28 (35.4)	36 (39.1)	26 (40)	12 (21.1)	9 (28.1)	111 (34.2)
Female	51 (64.6)	55 (59.8)	39 (60)	45 (79.9)	22 (68.8)	212 (65.2)
Prefer not to disclose or unknown	0	1 (1)	0	0	1 (3.1)	2 (0.5)
First-generation college student	40 (50.6)	49 (53.3)	50 (77)	42 (73.7)	25 (78.1)	226 (69.5)
Type of college or university currently enrolled in						
Public community college (grants associate’s degree only)	5 (17.9)	16 (57.1)	6 (21.4)	4 (14.3)	4 (14.3)	35 (10.8)
Public college or university	51 (21.3)	58 (24.1)	41 (17.1)	33 (13.4)	23 (9.6)	206 (63.4)
Private college or university	23 (22.5)	18 (17.7)	18 (17.7)	20 (19.6)	5 (4.9)	84 (25.8)
Years of college						
<1	0	10 (45.5)	9 (40.1)	0	3 (13.6)	22 (6.8)
1	36 (28.1)	33 (25.8)	30 (23.4)	17 (13.3)	12 (9.4)	128 (39.4)
2	41 (25)	42 (25.6)	26 (15.9)	38 (23.2)	17 (10.4)	164 (50.5)
3	2 (22.2)	5 (55.6)	0	2 (22.2)	0	9 (2.8)
4	0	2 (100)	0	0	0	2 (0.6)

Additionally, as outlined by the RWJF, most participants indicated an interest in medicine (143 [45.5%]), dentistry (76 [24.2%]), and pharmacy (35 [11.1%]) before their participation. A breakdown of prehealth interests (regardless of their selected pathways) before and after the program is given in Table 3.

**Table 3.** Participants Career Interests Before and After SHPEP

Career interest	No. (%) of participants
Pre-SHPEP (n = 314)	
Premedical	143 (45.5)
Predental	76 (24.2)
Prepharmacy	35 (11.1)
Pre–public health	24 (7.6)
Pre–physician assistant	14 (4.5)
Prenursing	9 (2.9)
Pre–veterinary medicine	3 (1.0)
Undecided	8 (2.5)
Other	2 (0.6)
Post-SHPEP (n = 249)	
Premedical	107 (43.0)
Predental	70 (28.1)
Prepharmacy	28 (11.2)
Pre–public health	16 (6.4)
Pre–physician assistant	12 (4.8)
Prenursing	6 (2.4)
Pre–veterinary medicine	3 (1.2)
Undecided	6 (2.4)
Other	1 (0.4)

Abbreviation: SHPEP, Summer Health Professions Education Program.

### Mixed Analysis of Variance Analysis

Pre-post barriers, as well as motivators, served as within-participant variables. Program year served as a between-participant variable. The analysis yields no evidence of an interaction effect associated with year and pre-post scores. Similarly, there were no statistically significant differences in pre-post scores based on program year. The analysis, however, yielded a significant within-participant difference before and after the program for motivators. Table 4 details the means (SDs) for perceived motivators and barriers before and after the program by year. On average, participants’ perceived motivators increased after program completion (mean [SD] *x̅* = 84.60 [9.67] vs 80.95 [8.93]; *P* = .001). Conversely, perceived barriers decreased between the beginning and end of the program (mean [SD] *x̅* = 48.02 [13.20] vs 51.72 [11.39]; *P* = .008). Effect sizes for the pre-post differences, measured via the Cohen *d,* were small. Pearson correlations between variables (Table 5) ranged from weak to moderate.

**Table 4.** Means (SDs) for Perceived Motivators and Barriers Before and After the Summer Health Professions Education Program by Year

Variable	Mean (SD) *x̅*			
	Preprogram motivators	Postprogram motivators	Preprogram barriers	Postprogram barriers
2017	81.66 (8.82)	86.36 (8.77)	51.24 (11.04)	48.80 (14.03)
2018	83.55 (8.74)	85.53 (11.01)	49.65 (12.43)	46.51 (14.30)
2019	79.75 (8.02)	80.98 (8.85)	54.22 (10.70)	50.57 (11.86)
2020	78.67 (7.93)	83.08 (7.76)	52.33 (10.54)	50.52 (7.97)
2021	78.25 (11.18)	85.05 (9.06)	52.69 (11.48)	42.55 (11.99)
Total	80.95 (8.93)^a^	84.60 (9.67)^a^	51.72 (11.39)^a^	48.02 (13.20)^a^

^a^ The correlation is significant at a significance level of *P* < .01.

**Table 5.** Pearson Correlations Between Perceived Motivators and Barriers Before and After the Summer Health Professions Education Program

Variable	Preprogram motivators	Preprogram barriers	Postprogram motivators	Postprogram barriers
**Preprogram motivators**				
*R*	1	−0.41^a^	0.58^a^	−0.33^a^
*P* value	NA	<.001	<.001	<.001
**Preprogram barriers**				
*R*	−0.41^a^	1	−0.31^a^	0.61^a^
*P* value	<.001	NA	<.001	<.001
**Postprogram motivators**				
*R*	0.58^a^	−0.31^a^	1	−0.46^a^
*P* value	<.001	<.001	NA	<.001
**Postprogram barriers**				
*R*	−0.33^a^	0.61^a^	−0.46^a^	1
*P* value	<.001	<.001	<.001	NA

Abbreviation: NA, not applicable.

^a^ The correlation is significant at a significance level of *P* < .001 (2-tailed).

## Discussion

Summer enrichment programs such as the SHPEP have been recognized as key to diversifying the health professions, likely contributing to a reduction in health disparities among underrepresented populations. Findings suggest that the SHPEP has been a successful interprofessional enrichment program that promotes perceived motivators and alleviates perceived barriers to entering and succeeding in health professional school.

By analyzing the program’s latent factors, we found that the enhancement program had a small to moderate significant influence on perceived barriers and motivators to entering health professional schools across a 5-year span. Because the SHPEP at the University of Florida was offered online in 2020 and 2021, the director was concerned about the program’s impact due to limited personal connections and hands-on experience. Many participants yearned for in-person interactions and shadowing experiences. Despite a shift to an online format and a decrease in the number of scholars who completed the survey, the SHPEP still had a positive impact on students and provided a supportive infrastructure during the pandemic. These findings are congruent with the analysis of other virtual summer enrichment programs offered during the pandemic: the University of Pennsylvania’s Penn Access Summer Scholars program found that students were more confident in their research skills, had a greater concept of physicians’ identity, felt better prepared to enter medical school, and strengthened their bonds with research mentors, program coordinators, and staff.^32^ Additionally, the Bronx Health Opportunities Partnership-Einstein Virtual Summer Program for underrepresented students in medicine improved many aspects of career self-efficacy for students, such as feeling confident in how to achieve career goals, network, interview, and write a resume.^20^ Our findings also support previous literature suggesting that summer enrichment programs have positive influences on students, such as improvements in research skills, research self-efficacy,^18^ academic knowledge, and academic self-efficacy.^19^

### Future Directions

Due to the lack of follow-up, the SHPEP must be evaluated further to determine its long-term influence on the health professional workforce for underrepresented populations. The low representation of American Indian or Alaskan Native students also warrants further investigation to better understand motivators and barriers for these individuals.

Although we did not find differences before and during the SHPEP COVID-19–related program delivery modifications, the virtual program may have added a layer of challenges for students. For example, students interested in medicine as a career were systemically limited by the pandemic due to reduced traditional in-person clinical training. Thus, many students may have experienced imposter syndrome for not having enough training experience.^33^ The limited career and educational opportunities, as well as discrimination and racism, faced by underrepresented individuals were also exacerbated due to the pandemic.^33^ COVID-19–related virtual programming may also make many students feel disconnected from school activities, accounting for the low response rates during 2020 to 2021. More examination of COVID-19 pandemic–related long-term impacts and outcomes (eg, mental health and program participation) is required to determine meaningful variables that may affect perceived barriers and motivators for underrepresented students interested in pursuing health professional degrees. Future work should also investigate the impact of the long-term connections made within pathway programs to identify curriculum components that contribute most to successful outcomes.

Additionally, studies will need to evaluate the impact of the recent decision on affirmative action by the US Supreme Court. Weaponized antidiversity initiatives will likely serve as structural determinants for preventing representation in the health care workforce. The recommendations from the Sullivan Commission’s *Missing Persons* landmark report^34^ are more important than ever. Policymakers, leaders, key stakeholders, community agents and organizations, public health agencies, primary and secondary schools, and higher education institutions should incorporate evidence-based interventions to promote interests in health education, remove barriers and improve motivators, and prepare underrepresented students to pursue and persevere in the health professional workforce.^34^ At the same time, understanding the political and social determinants of health are fundamental for upstream interventions to dismantle health inequity among underrepresented populations. More research on policy-relevant and action-oriented interventions must be conducted to reveal the detrimental impacts of health inequity for everyone, regardless of their identities.^35^

Perceived barriers and motivators are contextual factors that influence underrepresented students’ pathways into the health profession and the relationship between health profession career interests and choices.^36^ To increase representation and diversity within the health professions, applying effective strategies and multilayered approaches to improve students’ motivators and reduce barriers, as well as facilitate interest and entry into the health professions, is necessary.^37^ Knowledge from this study can assist educators, policymakers, and health care professionals who wish to implement similar enrichment programs. There is a strong need for innovative partnerships with underserved communities, requiring stakeholders to adopt strategies that demonstrate a strong commitment to increasing diversity in the health professions and develop viable funding mechanisms to support enrichment programs such as the SHPEP.

### Limitations

Our study has some limitations. The SCBS is an anonymous survey that relies on voluntary responses. Low response rates in 2020 and 2021 and selection and response bias are potential limitations. There is also a lack of alumni follow-up, specifically related to previous cohorts’ rates of entering graduate schools and pursuit of health careers. Additionally, American Indian or Alaskan Native individuals are not only underrepresented in health care professions but are also the smallest racial population in the SHPEP.

## Conclusions

This cohort study provides unique insights into the impact of the SHPEP at a large university in the southern US on addressing barriers and motivators for entry into the health professions among underrepresented students. By offsetting some of the challenges underrepresented students face, as well as providing self-efficacy tools to overcome barriers, enrichment programs can help produce health care professionals who are more likely to serve in underrepresented communities and work to reduce related health disparities.

## Notes

Supplement.
Data Sharing Statement
