# Supplementary material for: Perceived Success After Participation in the Summer Health Professions Education Program
Source: JAMA Netw Open. 2024 Jan 26;7(1):e2352440. doi: 10.1001/jamanetworkopen.2023.52440 (PMC10818219; doi:10.1001/jamanetworkopen.2023.52440)
Supplement: Supplement. — Data Sharing Statement [file jamanetwopen-e2352440-s001.pdf]

Data Sharing Statement

Xirau-Probert. Perceived Success After Participation in the Summer Health Professions Education Program. *JAMA Netw Open*. Published January 26, 2024.
doi:10.1001/jamanetworkopen.2023.52440

Data
Data available: No
